# Case report: Successful administration of cariprazine in a young, severely ill patient with recurrent relapses of schizophrenia and persistent negative symptoms

**DOI:** 10.3389/fpsyt.2023.1134692

**Published:** 2023-03-09

**Authors:** Jelena Vrublevska

**Affiliations:** Department of Psychiatry and Narcology, Riga Stradins University, Riga, Latvia

**Keywords:** cariprazine, schizophrenia, case report, antipsychotic combination, psychotic relapse

## Abstract

The present case report describes a young man diagnosed with schizophrenia and presents a “revolving door” (RD) phenomenon. He was hospitalized in an acute psychiatric clinic three times in 1 year. After each hospitalization, he was discharged with incompletely reduced psychotic symptoms, persistent negative symptoms, low functioning, lack of insight, and adherence. He had an insufficient response to maximally tolerated doses of antipsychotic monotherapy with haloperidol and risperidone. Moreover, his treatment was complicated due to the low accessibility of long-acting injectable atypical antipsychotics (LAI) in the country and his refusal of the only available atypical LAI paliperidone palmitate and refusal to take clozapine. Due to limited alternatives, the decision to administer combinations of antipsychotics was made. Since his diagnosis, he received several combinations of antipsychotics, i.e., haloperidol + quetiapine, risperidone + quetiapine, haloperidol + olanzapine, risperidone + olanzapine, but without sufficient clinical effectiveness. Although combinations of antipsychotics reduced his positive symptoms to some degree, persistent negative symptoms and extrapyramidal side effects were observed. After initiating cariprazine, which was combined with olanzapine, improvement in the patient’s positive symptoms, negative symptoms, and overall functioning was detected. The combination of medications mentioned above facilitated the therapeutic alliance, thus providing control over the symptoms and preventing psychiatric hospitalizations.

## Introduction

Schizophrenia is a complex, chronic, and debilitating disease with a worldwide lifetime prevalence of around 5 per 1,000 persons and an estimated heritability of around 0.64 (1, 2). The onset of schizophrenia, defined by the emergence of positive, negative, cognitive symptoms as well as decline in social and occupational functioning, significantly impairs the patients’ skills required for social integration and maintenance of personal support systems (3). Significant residual symptoms are highly prevalent even in remitted schizophrenia patients (4). The most frequent residual symptoms are negative and cognitive symptoms alongside with reduced positive symptoms (5–7). There is a significant association between the presence of residual symptoms and global functioning (8). In addition, the severity of residual symptoms is directly associated with a risk of relapse and poor outcome (7). Importantly, relapse is one of the most significant barriers to recovery and rehabilitation, resulting in great distress and poor quality of life (9–11). Patients with schizophrenia spectrum disorders often relapse even when they are undergoing treatment (12). Furthermore, the first 2–5 years are believed to be a key regarding the long-term functional and clinical prognosis associated with schizophrenia (13).

There is a subpopulation of schizophrenia patients referred to as RD patients due to the frequency of readmissions in psychiatric units. The phenomenon has various definitions. RD indicates repeated hospitalizations of patients unable to sustain an independent life in the community. It was found that 12% of patients with schizophrenia meet RD criteria defined as more than three admissions in 30 months (14, 15). The authors have indicated that young males are at higher risk for RD, as well as higher scores on measures of psychosis and noncompliance with medication were found to be strong predictors for RD (14, 16). Additionally, patient-related factors as low social support, low functioning, low education level, unemployment, and living alone are determinants of frequent re-hospitalization (17). Environmental factors may also influence this phenomenon: the “urban living,” and family conflicts, may increase re-hospitalizations (18, 19). One of the proposed criteria is defined as three or more hospitalizations in 18 months or two or more hospitalizations in 12 months in the presence of clozapine use (15).

Evidence-based treatments for schizophrenia exist, nonetheless they have limitations. Antipsychotic medications are associated with a wide range of side effects, such as extrapyramidal symptoms, sedation, increased prolactin levels, weight gain, and cognitive impairment (20). These side effects caused by antipsychotics therefore have a negative impact on adherence (21). In addition, patients who had experienced any side effects tend to doubt the effectiveness of the antipsychotic (22).

Cariprazine is a third-generation antipsychotic medication that received approval by the Food and Drug Administration (FDA) for the acute treatment of schizophrenia in 2015 and for the maintenance treatment of schizophrenia in 2017. FDA also approved cariprazine for depressive (in 2019) and acute manic and mixed episodes associated with bipolar I disorder (in 2015) and as adjunctive treatment to antidepressant therapy in major depressive disorder (in 2022) (23, 24). Cariprazine was approved by the European Medicines Agency for the treatment of schizophrenia in adult patients in 2017 (25). Cariprazine has a different receptor profile from the other antipsychotic medications, as it is a dopamine D3-D2 receptor partial agonist with preferential binding to the D3 receptors (26). Data from short- and long-term double-blind, placebo-controlled studies support the recommendation of cariprazine as a safe and effective treatment for adult patients with acute schizophrenia (27–30). In addition, several studies including observational studies showed its effectiveness in the treatment of negative and cognitive symptoms as well (27, 29, 31). Nonetheless, in case of new medications, it is particularly important to generate more data on both effectiveness and tolerability as it can provide valuable information. It is of interest to obtain more clinical information on the effect of cariprazine on adherence and RD phenomenon.

In this article, a clinical case of a young man with schizophrenia is described who was hospitalized three times within 1 year and who had low adherence to antipsychotic treatment, displayed residual symptoms of schizophrenia and low global functioning. The patient provided written informed consent for the publication of this report.

## Case presentation

A 22-year-old male with a recent diagnosis of schizophrenia has been hospitalized three times within 1 year despite antipsychotic treatment with haloperidol, risperidone, and combinations of antipsychotics (treatment overview during hospitalizations and outpatient treatment is presented in Tables 1, 2). Due to the different antipsychotics, the patient experienced extrapyramidal side effects in a form of Parkinsonism, had residual positive and prominent negative symptoms, and presented a low level of psychosocial functioning. The patient also showed low adherence in-between hospitalizations and never visited the outpatient clinic. After the third hospitalization, outpatient treatment regimens with different antipsychotic combinations were tried, nonetheless without sufficient success. Therefore, a switch to cariprazine (6 mg/day) + olanzapine (10 mg/day) combination was initiated which resulted in considerable improvement in both positive and negative symptoms as well as in overall functioning.

**Table 1:** Medication history overview (inpatient episodes).

Medication/s	Days taking medication/s	CGI-S	CGI-I	Comment
First hospitalization (03/2020)				
HAL up to 15 mg/d	25 days	6	3	Combination with Diazepam reduced anxiety and partially improved positive symptoms but caused extrapyramidal side effects. THP was administered.
RIS 4 mg/d	60 days	5	3	HAL was switched to RIS. Patient stopped taking it due to the parkinsonism and lack of compliance.
Second hospitalization (04/2021)				
HAL up to 15 mg/d + QUE 50 mg/d	14 days	5	4	Combined with Diazepam improved symptoms of anxiety and partially improved psychotic symptoms. THP was administered to manage parkinsonism.
HAL 15 mg + OLA 10 mg/d	4 days	5	3	QUE was switched to OLA due to anxiety and positive symptoms.
HAL 7.5 mg/d + OLA 20 mg/d	6 days	4	3	Some improvement in positive symptoms. The patient requests discharge from the hospital.
Third hospitalization (05/2021; 2 week after previous discharge)				
ARI 15 mg	1 day	5	6	Patient refused to continue with it and stated that it increases anxiety and worsens bodily sensations.
HAL up to 15 mg/d + OLA 20 mg/d	13 days	6	3	Combination of Haloperidol and Olanzapine with Diazepam and THP partially reduced anxiety and positive symptoms. The patient requests discharge from the hospital. After discharge stopped taking medications.
HAL 7.5 mg/d + OLA 20 mg/d	8 days	5	3	

HAL, haloperidol; RIS, risperidone; QUE, quetiapine; OLA, olanzapine; ARI, aripiprazole; THP, trihexyphenidyl; CGI-S, Clinical Global Impression Scale – Severity; CGI-I, Clinical Global impression Scale – Improvement.

**Table 2:** Timeline summary of patient events and medication across the outpatient treatment period.

Medication/s	Days taking medication/s	CGI-S	CGI-I	Comment
Outpatient care 06/2021				
RIS 6 mg/d	14 days	5	4	Restless, tensed, complains of anxiety and unpleasant sensations in the region of the heart, and the influence on the internal organs.
RIS 6 mg/d + OLA 20 mg/d	20 days	5	4	Anxiety, discomfort in the area of the heart, sense of the influence on internal organs decreased. Feels drowsy. Negative symptoms and psychomotor retardation.
Outpatient care 07/2021				
RIS 6 mg/d + OLA 20 mg/d + QUE 50 mg/d	40 days	5	4	The patient’s mental state is unstable. Manifestation of thought insertion, increase in anxiety and worsening of senestopathies in the chest.
Outpatient care 09/2021				
RIS 6 mg + OLA 20 mg + Buspirone 30 mg/d Alprazolam available to reduce anxiety and irritability.	35 days	5	3	The patient was dissatisfied with QUE. At the visit is taciturn, irritable, and stingy in his answers. Positive symptoms. Increase of prolactin.
Outpatient care 10/2021				
OLA 20 mg/d + RIS down-titration and CAR up-titration to 6 mg/d + Buspirone 30 mg/d Alprazolam available to reduce anxiety and irritability.	34 days	5	3	The patient is apathetic, in an irritable mood, lives a closed lifestyle, and avoids social communication. Episodic thought insertion and somatic hallucinations in the chest persist.
Outpatient care 11/2021				
CAR 6 mg/d + OLA 20 mg/d + Buspirone 30 mg/d	30 days	4	3	Positive symptoms lessened. More energy, has become more active.
Outpatient care 12/2021				
CAR 6 mg/d + OLA 20 mg Down-titration of Buspirone	35 days	4	2	The patient’s mental state improves, he has become more active, goes outside the house, and reads. Improvement in negative symptoms. Residual positive symptoms.
Outpatient care 01/2022				
CAR 6 mg/d + OLA 10 mg/d	28 days	3	2	Improvement in social functioning. Episodic mild anxiety.
Outpatient care 02/2022				
CAR 6 mg/d + OLA 10 mg/d	35 days	3	2	Part-time employment. Mild negative symptoms. Rare mild anxiety. Prolactin is within a norm.

HAL, haloperidol; RIS, risperidone; QUE, quetiapine; OLA, olanzapine; CGI-S, Clinical Global Impression Scale – Severity; CGI-I, Clinical Global Impression Scale – Improvement.

## Background history

The patient had a family history of schizophrenia as his paternal grandmother had this diagnosis. Early developmental progression through predictable developmental phases was without any delay. The patient started school in time; however, his achievements were mediocre. His mother described him as a loner and withdrawn in relation to emotional expression. The first significant changes were noticed when the patient was 19 years old as he started complaining to have frequent headaches. At that time, the onset of unusual behavioral symptoms and markedly peculiar behavior occurred. He also became withdrawn, and had impairment in personal grooming. Two months prior to his first hospitalization, the patient lost his job, and started wandering. Frank psychotic symptoms manifested 1 week before psychiatric hospitalization.

The patient lives with his mother and has never been in a romantic relationship. He does not consume alcohol or other addictive substances. No clinically significant head injuries, chronic somatic illnesses nor allergic reactions are reported.

## Interventions and their outcome

An overview of three hospitalizations, administered medications, and comments about patient response to treatment is represented in Table 1. At the time of the first hospitalization, the patient’s clinical picture was characterized by disorganized thoughts and behavior, thought insertion, delusions of persecution and influence, and somatic hallucinations. Negative symptoms were also a source of his general dysfunction. According to the Structured Clinical Interview for DSM Disorders (SCID-CT), schizophrenia was diagnosed (32). Moreover, using the International Statistical Classification of Diseases and Related Health Problems 10th Revision (ICD 10) paranoid schizophrenia (F20.0; ICD-10) was detected (33). Any organic causes of symptoms due to brain damage or somatic disease, psychoactive substance-use disorders, and affective disorders were ruled-out. Diagnostic workup to exclude non-psychiatric etiology of the described symptoms involved overall including neurological evaluation, blood tests (hemogram, biochemistry, thyroid function, hepatitis B and C, syphilis and HIV serology, and vitamin B12), and urine drug screening (cannabinoids, amphetamines, cocaine, opioids). The results produced no abnormalities. A cranial CT, electroencephalogram, and electrocardiogram revealed normal results.

Assessment with the Positive and Negative Syndrome Scale (PANSS) (34) indicated a score of 48 in Marder positive PANSS subscale, a score of 37 on the Marder negative PANSS subscale, a score of 22 on the Marder cognitive PANSS subscale, a score of 20 on Marder anxiety/depression PANSS subscale, and a score of 20 on Marder hostility PANSS subscale. According to the Clinical Global Impressions Scale (CGI-S) (35–37), the patient was classified as severely ill (CGI-S = 6).

At the psychiatry inpatient unit, the patient received haloperidol up to 15 mg (which was initially given intramuscularly up to 15 mg/day in combination with intramuscular diazepam up to 20 mg/day. Since drug-induced parkinsonian symptoms appeared), trihexyphenidyl 6 mg/day was prescribed due to this adverse effect (38). Haloperidol was switched to risperidone 4 mg/day due to adverse effect. The patient remained hospitalized for 37 days, and was discharged on request. He took risperidone for 60 days and then discontinued due to lack of adherence.

After discharge from the psychiatric clinic, he did not visit a psychiatrist, did not work, lived a secluded life, and was socially isolated.

The patient was hospitalized for the second time 1 year later. At the admission, he had hallucinatory voices, delusions, somatic hallucinations, and agitation. Additionally, the patient showed diminished emotional expression, and asociality. In the inpatient unit, he was reinstated on treatment with haloperidol 15 mg/day in combination with diazepam up to 30 mg/day, and add-on a low dose of quetiapine 50 mg at bedtime for insomnia, and 6 mg/d of trihexyphenidyl due to emerge of drug-induced parkinsonism. After 2 weeks, quetiapine was switched to olanzapine 10 mg/day with a target dose of 20 mg/day for controlling positive, negative symptoms and to reduce anxiety and insomnia (39). The patient refused to have treatment with clozapine because of his suspiciousness. He was discharged on 24th day after admission with some improvements in positive symptoms but with existing negative symptoms and incomplete insight of his condition.

Finally, the patient was re-hospitalized for the third time within a week after his previous discharge due auditory pseudohallucinations, somatic hallucinations, and anxiety. The patient still did not agree to treatment with clozapine, explaining that this medication has many side effects. Aripiprazole 15 mg was attempted, but the patient refused to take it after 1 day due to an increase in anxiety and somatic hallucinations. As the previous combination of haloperidol 15 mg/day and olanzapine 20 mg/day was used with some success during the last hospitalization, a decision to return to that regime was made. Symptoms of anxiety were managed by add-on diazepam 15 mg/day. The patient requested discharge from the hospital after 22 days and stopped taking medication.

One month after the third hospitalization, outpatient treatment was finally initiated. A timeline summary of patient events and medication across the outpatient treatment period is presented in Table 2. During the first outpatient visit, the assessment with the PANSS showed a score of 33 in the Marder positive subscale, a score of 31 in the Marder negative subscale, a score of 16 in the Marder cognitive subscale, a score of 15 in the Marder anxiety/depression subscale, and a score of 14 in the Marder hostility subscale. As the patient already tried different antipsychotic medications, refused clozapine and long-acting injection (paliperidone palmitate), the decision was to start a risperidone 6 mg/day. The patient had partial response in terms of alleviation of positive symptoms and anxiety. On day 14, risperidone was combined with olanzapine 20 mg/d because of its sedative effects (40). This combination did not cause any extrapyramidal side effects. As the patient continued to have sleep disturbances and anxiety in the evenings, the patient was given an add-on quetiapine 50 mg at bedtime (41). Within 40 days of treatment no significant improvement was achieved. Quetiapine was discontinued, and anxiety symptoms were alleviated with alprazolam (up to 3 mg/day), and buspirone (30 mg/day). Residual psychotic symptoms, anxiety symptoms, as well as negative and cognitive symptoms persisted in the background despite of the therapy. The results of blood analysis produced increased prolactin level. The decision was taken to switch from risperidone to cariprazine. Cariprazine was prescribed in a dose 1.5 mg/day, and was increased every second day up to 6 mg/day (42). Tapering of risperidone was initiated on day 10 after starting cariprazine. The patient responded well both in terms of tolerability and alleviation of positive and improvement of negative symptoms. After 64 days of cariprazine administration, buspirone was gradually discontinued. The patient stabilized regarding his positive symptoms and anxiety; therefore, olanzapine was down-titrated to 10 mg/day. With the present combination the patient reported interest to be employed, resumed communication with acquaintances, and started outdoor physical activities. The results of blood analysis with metabolic profile and prolactin levels produced no abnormalities.

**Figure 1:**
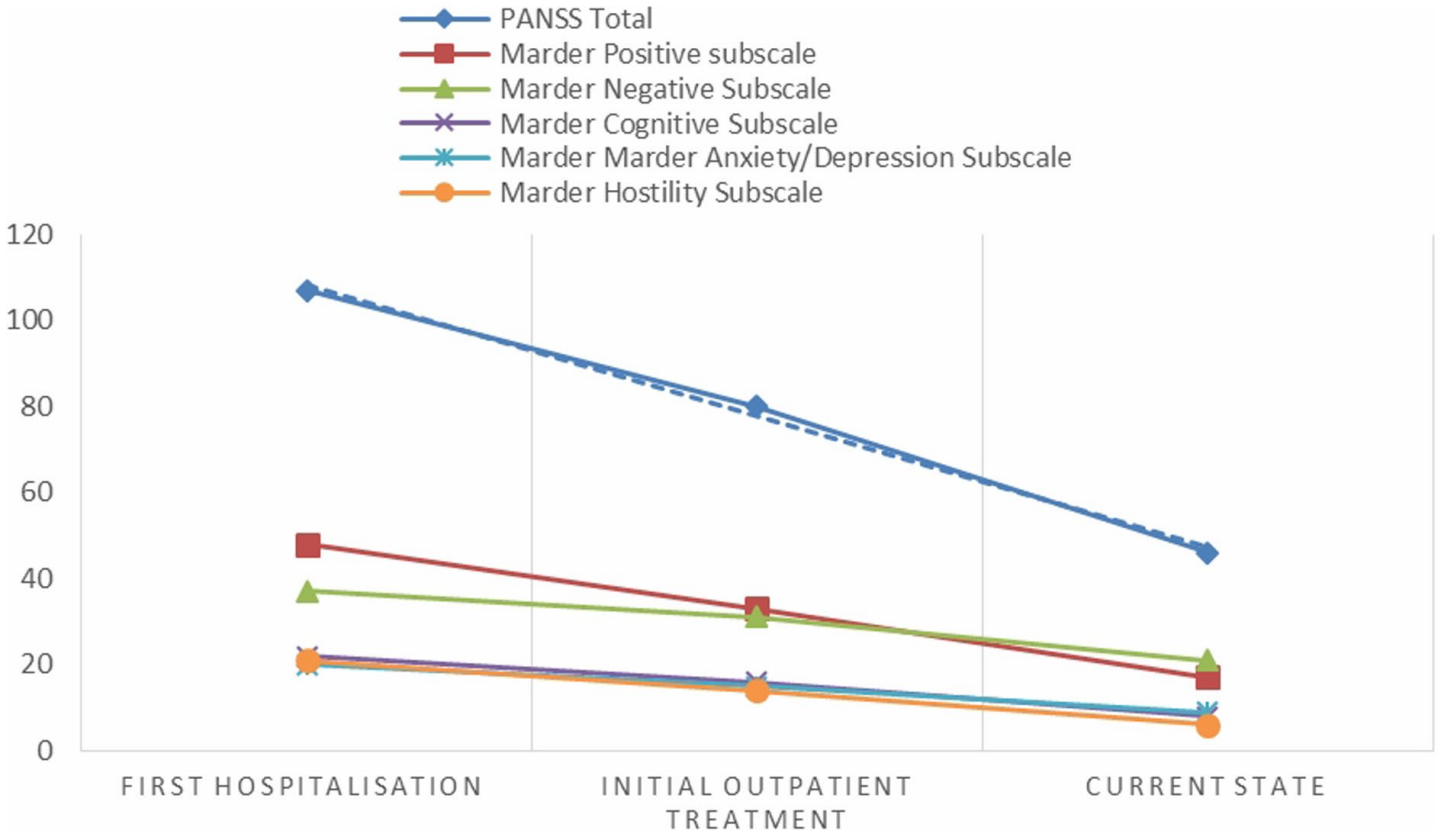
PANSS Marder and total scores at different stages of patient assessment.

Four months after add-on cariprazine 6.0 mg/day, the patient found work, where he is still employed part-time. He visits a psychiatrist on a regular basis. To date, his condition is stable, however, he has episodic anxiety and sleep maintenance problem. For these reasons, he still continues to take cariprazine 6.0 mg/day in combination with olanzapine 10 mg/day. Importantly, the patient does not have any extrapyramidal side effects, nor metabolic changes, and his body mass index and prolactin levels are within the normal range. In general, his negative symptoms improved, but he still has a partially diminished ability to express emotions, slight reduction of verbal fluency, and reduced initiation and persistence in goal directed behavior. The assessment with the PANSS revealed that there was an improvement in all Marder factors of the PANSS: a score of 17 on the Marder positive subscale, a score of 21 on the Marder negative subscale, a score of 8 on the Marder cognitive subscale, a score of 9 in Marder anxiety/depression subscale, and a score of 6 in Marder hostility subscale. The total score of the PANSS dropped from 158 (first hospitalization) to 66 (4 months after add-on cariprazine 6 mg/day). The scores of all five Marder dimensions of the PANSS and total PANSS score subscales during the patient’s first admission to the psychiatric clinic, during the first assessment in the outpatient department, and after administering cariprazine are presented in Figure 1.

## Discussion

This case report describes a ‘difficult to treat’ young patient who was diagnosed with schizophrenia at the age of 22 and who had three hospitalizations due to worsening of symptoms during 1-year period. After each hospitalization, he was discharged having some positive and prominent negative symptoms. The course of the disorder was affected by the extrapyramidal side effects of antipsychotic medications, as well as lack of insight of the illness and adherence to the treatment. Moreover, the treatment process was complicated by the patient’s refusal of clozapine and long-acting formulation injections. Since his diagnosis, he received several combinations of antipsychotics without appreciably sufficient clinical effectiveness.

When evaluating the course of the patient’s illness, it is evident that negative symptoms were prevalent already in the premorbid period of the disorder which was then followed by the acute exacerbation of psychotic symptoms. After initiating cariprazine 6 mg/day, which was combined with olanzapine, improvement in the patient’s positive symptoms, negative symptoms, and overall functioning was detected. Improvement in the adherence to treatment was also achieved.

In the treatment of his first acute episode of schizophrenia which was accompanied by agitation, typical antipsychotic and benzodiazepine were reasonably prescribed. The patient experienced adverse effect in the form of Parkinsonism which then possibly resulted in decreased adherence to any future treatment. Besides adverse effects, there were several other factors that worsened the adherence and made the treatment especially challenging. These were the insufficient effectiveness of antipsychotics, lack of insight, refusal of treatment with clozapine, persisting psychotic symptoms, prominent negative symptoms, and social withdrawal leading to a poorer outcome, including an increased risk of relapse, RD phenomenon and decreased quality of life (22).

It is important to note that approximately one-third of patients with schizophrenia have a poor response to antipsychotic medications (43), therefore combinations of antipsychotics are frequently considered. Main reasons for initiating antipsychotic polypharmacy treatment include general aspirations to improve, expand and maintain treatment efficacy, as well as a preventive measure against relapse and rehospitalization in relation to outcome (44). Inadequate response to antipsychotic monotherapy and prominent negative symptomatology are significant reasons for initiating antipsychotic combination treatment in severely ill patients (44–46).

A 16-week observational study in real-life settings examining the effectiveness and safety of cariprazine in schizophrenia outpatients with negative symptoms reported that about half of the patients continued to take the antipsychotic medication with cariprazine that was secondary to their previous antipsychotic. The results of the study demonstrated significant improvement in negative symptoms though and overall functioning. Olanzapine was one of the most used antipsychotic medications (31).

The combination of cariprazine and olanzapine may mimic the complementarity of the neuroreceptor target profiles of clozapine and aripiprazole (44). Worth mentioning, the combination of D2 partial agonist aripiprazole and clozapine in improvement of negative symptoms was found to be superior in comparison to clozapine alone (47). Additionally, it provides the strongest protection against rehospitalization (48). From a pharmacodynamic profile point of view, it is reasonable to assume that the potent partial agonist properties of D2 and D3 of cariprazine would result in a therapeutically beneficial combination by complementing olanzapine’s relative lack of strong interactions with these targets (44).

With regard to adverse effects, no extrapyramidal side effects were observed, and prolactin levels normalized. The patient had parkinsonism during the previously prescribed therapy, whereas no such adverse event occurred when he was switched to cariprazine. *Post hoc* pooled analyses of data from two long-term open-label studies indicate that treatment with cariprazine is generally well tolerated, suggesting low level of discontinuation due to extrapyramidal side effects (49). Results from randomized, double-blind, placebo-controlled studies indicate that the largest decreases in prolactin following cariprazine treatment were observed in patients who were previously taking medications with a high risk of elevating prolactin, suggesting that cariprazine treatment was associated with normalization of prolactin levels in these patients (50). There did not appear adverse effects on metabolic variables, weight and ECG.

Change from the first hospitalization in 2020 to the current state was observed in the total PANSS score by 92 points. Moreover, the patients’ improvement was observed in all five PANSS Marder factors. Improvement as measured by the CGI-S was observed after 60 days of starting the combination of cariprazine and olanzapine. Changes in the dynamics of the patient’s mental state are in accordance with cariprazine efficacy studies (51). From an efficacy perspective, it appears probable that combination of cariprazine and olanzapine can be recommended to patients with prominent and persistent negative symptoms, and those with residual positive symptoms (44).

Given the history of the patient, it is a significant achievement that there have been no hospitalizations after administering cariprazine add-on treatment to olanzapine. Finally, it is highly important to involve patients in treatment decisions such as choosing the right medications. To do this, one should focus on good communication and shared decision making. This way an initial negative experience of the inpatient can be avoided (52).

## Limitations

An important limitation of this article is that it is based on the description of the case of one patient, which limits generalization to a wider patient population. There is no sufficient information available, whether structured psychoeducation, explaining the role of each medication and possible side effects was performed during the hospitalizations.

## Conclusion

Several risk factors for RD were observed in the patient, such as high scores of psychosis detected by the PANSS, lack of adherence to the treatment, residual symptoms, and low functioning. Cariprazine as add-on to olanzapine treatment improved the patient’s mental state in terms of improvement on the PANSS total score and on all five PANSS Marder factors. The above-mentioned combination of medications facilitated the patient’s adherence to treatment as well as therapeutic alliance, thus providing control over the symptoms and preventing psychiatric hospitalizations.

## Patient perspective

The patient positively evaluates the current course of treatment, although he refuses the proposed psychosocial interventions, claiming that he will be able to maintain his health on his own. He is hopeful of returning to full-time work.

## Data availability statement

The raw data supporting the conclusions of this article will be made available by the author, without undue reservation.

## Ethics statement

Ethical review and approval was not required for the study on human participants in accordance with the local legislation and institutional requirements. The patients/participants provided their written informed consent to participate in this study. Written informed consent was obtained from the participant/patient(s) for the publication of this case report.

## Author contributions

JV contributed to conception of the manuscript and wrote and edited the manuscript.

## Funding

This study received funding from Gedeon Richter Plc. Representative office in Latvia. The funder was not involved in the study design, collection, analysis, and interpretation of data, the writing of this article or the decision to submit it for publication.

## Conflict of interest

The author declares that the research was conducted in the absence of any commercial or financial relationships that could be construed as a potential conflict of interest.

## Publisher’s note

All claims expressed in this article are solely those of the authors and do not necessarily represent those of their affiliated organizations, or those of the publisher, the editors and the reviewers. Any product that may be evaluated in this article, or claim that may be made by its manufacturer, is not guaranteed or endorsed by the publisher.
